# Invasive yeast infection in paediatric acute lymphoblastic leukaemia: A report from the multi‐international clinical trial AIEOP‐BFM ALL 2009

**DOI:** 10.1111/bjh.70405

**Published:** 2026-02-24

**Authors:** Andreas H. Groll, Simone Cesaro, Julia Alten, Andishe Attarbaschi, Draga Barbaric, Daniel Ebrahimi‐Fakhari, Nicole Bodmer, Valentino Conter, Sarah Elitzur, Anja Möricke, Martin Schrappe, Jan Stary, Ester Zapotocka, Martin Zimmermann, Thomas Lehrnbecher

**Affiliations:** ^1^ Infectious Disease Research Program, Department of Pediatric Hematology and Oncology and Center for Bone Marrow Transplantation University Children's Hospital Münster Münster Germany; ^2^ Paediatric Haematology Oncology, Department of Mother and Child Azienda Ospedaliera Universitaria Integrata Verona Italy; ^3^ Pediatric Hematology‐Oncology University Hospital Schleswig‐Holstein, Campus Kiel Kiel Germany; ^4^ St. Anna Children's Hospital Medical University of Vienna Vienna Austria; ^5^ St. Anna Children's Cancer Research Institute Vienna Austria; ^6^ Sydney Children's Hospital Randwick New South Wales Australia; ^7^ University Children's Hospital Zurich Zurich Switzerland; ^8^ Tettamanti Center Fondazione IRCCS San Gerardo dei Tintori Monza Italy; ^9^ Schneider Children's Medical Center and Gray Faculty of Medical and Health Sciences Tel Aviv University Tel Aviv Israel; ^10^ Department of Oncology St Jude Children's Research Hospital Memphis Tennessee USA; ^11^ Czech Working Group for Pediatric Hematology Prague Czech Republic; ^12^ Department of Pediatric Hematology/Oncology University Hospital Motol Prague Czech Republic; ^13^ 2nd Faculty of Medicine Charles University Prague Czech Republic; ^14^ Department of Pediatric Hematology/Oncology Hannover Medical School Hannover Germany; ^15^ Divison of Pediatric Hematology and Oncology, Department of Pediatrics Johann Wolfgang Goethe‐University Frankfurt Germany

**Keywords:** cancer, *Candida*, children, epidemiology, leukaemia, mycoses, outcome, yeast

## Abstract

Prospective data of a large randomized multi‐international leukaemia trial revealed an incidence of 1.1% of invasive yeast infections (IYIs) in children with acute lymphoblastic leukaemia. IYIs occurred predominantly during induction chemotherapy, presented as bloodstream infection ± tissue involvement, were due to a variety of yeast and had an attributable mortality of 5.9%.

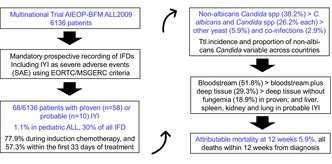

To the Editor,

Invasive fungal diseases (IFDs) remain important causes for infectious morbidity in immunocompromised children and adolescents with a negative impact upon overall survival.^1^, ^2^ Regrettably, however, most analyses of IFDs in paediatric patient populations at risk are limited by their restriction to individual centres, differences in IFD definitions, population denominators and fungal pathogens included, and systematic large‐scale data on the incidence, presentation and outcome of IFDs in defined paediatric patient populations at risk are lacking.^3^ Nevertheless, such data are important for recognizing specific risk settings and for improving prophylaxis, diagnostics and management of established infections.

Contemporary multinational epidemiological data on incidence, presentation and outcome of invasive yeast infections (IYIs) in paediatric patients with newly diagnosed acute lymphoblastic leukaemia (ALL) are scarce. We therefore analysed prospectively captured proven/probable IYI of children enrolled in the international, multi‐centre clinical trial AIEOP‐BFM ALL 2009.

Details of the total study population, treatment modalities and data collection have been previously reported.^2^ In brief, a total of 6136 children with ALL enrolled upon diagnosis in the international, multicentre, prospective randomized Phase III clinical trial AIEOP‐BFM ALL 2009 (EudraCT 2007‐004270‐43) were included in the analysis. The study was performed in seven countries (Australia, Austria, Germany, Israel, Italy, Switzerland and the Czech Republic) and open for enrolment between 1 June 2010 and 28 February 2017. The study was approved by the appropriate national and local review boards and was conducted in accordance with the Declaration of Helsinki and the applicable national laws. Informed consent was obtained from the parents or guardians of each patient included in the study, as required by ethical standards and national guidelines.

IFDs were considered a serious adverse event of special interest, and as such, their prospective reporting by the participating institutions was mandatory. IYIs were categorized as proven or probable according to the consensus definitions of the European Organization for Research and Treatment of Cancer and the Mycoses Study Group Education and Research Consortium,^4^ and yeast isolates were named according to recently proposed global consensus guidelines for nomenclatural designation.^5^ Patient data were retrieved from the AIEOP‐BFM database. Missing data were claimed from the participating centres, and conflicting results were solved by direct contact and discussion. In case a patient experienced multiple IFDs over time, only the first episode was included in the analysis.^2^ Statistical analysis was performed using SPSS 29.0 (IBM, USA). Comparisons were performed by chi‐squared or Fisher's exact test, where applicable, with *p*‐values <0.05 considered statistically significant.

Among 6136 children and adolescents enrolled into AIEOP‐BFM ALL 2009, 68 episodes of proven or probable IYIs were reported^2^ (34 females/34 males, median age [range] 4.6 years [1.1–17.9]), accounting for an incidence rate of 1.1% (range per country 0%–2.7%) and a relative proportion of 29.2% among all IFDs reported in the trial. Fifty‐eight patients had precursor B‐lymphocyte ALL (B‐ALL), 10 precursor T‐lymphocyte ALL (T‐ALL) and three patients had *Down* syndrome. Treatment allocation was to the standard‐risk (SR) arm in 17 (25%), the intermediate‐risk (MR) arm in 28 (41.2%) and the high‐risk (HR) arm of the trial in 23 (33.8%) patients respectively. Whereas no differences were found between patients with precursor B‐ALL and T‐ALL (10/871 [1.1%] vs. 58/5233 [1.1%]; *p* = 0.86) and for patients with *Down* syndrome (3/68 [4.4%] vs. 151/6127 [2.5%]; *p* = 0.23), the risk of IYIs was significantly higher in patients stratified as HR compared to SR or MR (23/1403 [1.6%] vs. 45/4764 [0.9%]; *p* < 0.05). IYIs predominantly occurred during the induction and consolidation chemotherapy of protocol I (77.9%), preferentially within the first 33 days after start of treatment (protocol IA; 57.3%). Infection prevalence during re‐intensification (protocols II and III; 13.3%), HR cycles (HR I, II or III; 5.9%) and protocol M (2.9%) were considerably lower.

There were 58 episodes of proven and 10 episodes of probable IYIs (Table 1). Proven infections were due to *Candida albicans* (18), non‐albicans *Candida* spp. (26), albicans/non‐albicans *Candida* co‐infection (2), unspecified *Candida* spp. (9) and other rare yeast organisms (3). Co‐infection with *Aspergillus* spp. was found in three of the 58 episodes. There was a predominance of non‐albicans *Candida* spp. in all countries except for Germany, and *C. tropicalis* and *C. parapsilosis* were the most frequently isolated non‐albicans *Candida* spp. Proven infections included isolated infections of the bloodstream (30; 51.8%), the bloodstream and various deep tissue sites (17; 29.3%) and deep tissue infections without fungaemia (11; 18.9%). Involvement of the bloodstream was most common (47; 81%), followed by the liver (13; 22%), the skin (9; 16%, embolic), the central nervous system (7; 12%), the lung (6; 10%) and various other sites. Probable infections were due to *Candida*‐ (9) and *Malassezia* (1) spp. and involved the liver (9), the spleen (6), the kidney and the lung (2 each) in various combinations (Table 1).

**TABLE 1:**
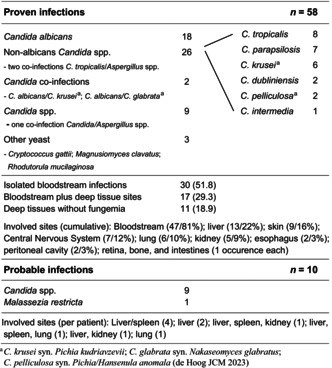
Characteristics of proven and probable invasive yeast infections in 68 patients enrolled into AIEOP‐BFM ALL 2009.

Infection‐related mortality at 12 weeks post‐diagnosis was 5.9% and similar at 1 year, accounting for an overall mortality from IYI of <0.1% (Table 2). All four deaths occurred within 6 weeks after the start of anti‐leukaemic therapy and were due to uncontrolled sepsis with multiorgan failure, with three of the patients being in documented cytomorphological remission at the time of death.

**TABLE 2:** Outcome of proven and probable invasive yeast infections in 68 patients enrolled into AIEOP‐BFM ALL 2009.

	Infection‐related mortality (*n* patients)	
	12 weeks	1 year
Proven infection (*n* = 58)	4	4
*Candida albicans* (*n* = 18)	1	1
Non‐albicans *Candida* spp. (*n* = 26)	1^a^	1^a^
Other yeast (*n* = 3)	2^b^	2^b^
Probable infection (*n* = 10)	0	0

^a^ *C. tropicalis*.

^b^ *Magnusiomyces clavatus* and *Cryptococcus gattii*.

Our analysis of prospectively captured data of AIEOP‐BFM ALL 2009 demonstrates an incidence rate of IYIs of 1.1% in children receiving treatment for de novo ALL with a relative proportion of 29.2% among all IFDs reported. IYIs occurred predominantly during induction therapy, presented mostly as bloodstream infections with or without tissue involvement, were due to a variety of different yeasts and had an attributable mortality rate of 5.9%.

While incidence and relative proportion of IYIs were in the same order of magnitude as reported in recent retrospective single‐centre or national studies in comparable healthcare settings,^6^, ^7^, ^8^, ^9^ their predominant occurrence during induction chemotherapy is notable and calls for consequences regarding systemic antifungal prophylaxis. As important risk factors such as prolonged, profound granulocytopenia, therapeutic use of glucocorticosteroids, presence of central venous catheters and treatment with broad‐spectrum antibacterial agents^3^, ^10^, ^11^, ^12^ are similarly prevalent in later treatment phases, prolonged periods of granulocytopenia in the weeks prior to diagnosis of leukaemia and acute comorbidities in this phase of treatment may account for this striking accumulation during induction chemotherapy.

Similar to the current paediatric literature in general^3^, ^12^ and European data from paediatric cancer patients,^13^ non‐albicans *Candida* spp. accounted for the majority of infections. Simultaneous (co‐) infections by two different *Candida* species or *Candida*‐ and *Aspergillus* species, and infections by so‐called ‘rare yeast’, which pose obvious therapeutic challenges, occurred in 7.4% and 5.9%, respectively; nine each (26.5%) of the proven and of the probable *Candida* infections remained without species identification and in vitro resistance testing and received calculated antifungal therapy only. In addition, more than 50% of patients had deep tissue involvement with liver, skin, central nervous system and the lung being affected in ≥10% of patients, which needs to be considered in the workup of the individual patient as well as in the selection and dosing of antifungal drug therapy. These findings highlight the diagnostic and therapeutic complexity of IYIs in children and adolescents with ALL and support the quest for specialized infectious disease supportive care.^14^

Infection‐related mortality due to IYI was 5.9% and similar to the 4.3% and 10% reported in two contemporary retrospective series from paediatric cancer patients in comparable healthcare settings^7^, ^8^ but lower than the accumulated historic experience of 10%–25%.^3^, ^12^ All attributable deaths occurred within 6 weeks from diagnosis of ALL during induction therapy, highlighting the call for appropriate antifungal prophylaxis during induction chemotherapy.

Most of the more recent epidemiological analyses on IFDs in paediatric cancer patients are monocentric and limited by differences in inclusion criteria, population denominators, IFD definitions and differentiation of fungal pathogens.^3^ In contrast, the current analysis is based on a very large, homogenous patient population of children and adolescents with de novo ALL enrolled on an international leukaemia trial with mandatory reporting of proven and probable IFDs, providing a solid denominator, standard IFD definitions and high data quality. Nevertheless, our study also has certain limitations. First, the study protocol did not mandate the reporting of antifungal prophylaxis, which clearly has an impact on the risk and the epidemiology of IFDs. Second, as diagnostic strategies were not standardized per protocol, they may have been different across centres and may have influenced the diagnosis of proven and probable IFDs. However, independent of these limitations, this uniquely systematic study provides important data on the epidemiology of IYIs in paediatric ALL patients in high‐income countries with full access to state‐of‐the‐art medical care and may help to refine their prevention, recognition and control in this vulnerable population.

## AUTHOR CONTRIBUTIONS

Andreas H. Groll, Thomas Lehrnbecher, Anja Möricke, Sarah Elitzur and Martin Zimmermann designed the research; Andreas H. Groll, Thomas Lehrnbecher, Simone Cesaro, Julia Alten, Andishe Attarbaschi, Draga Barbaric, Nicole Bodmer, Valentino Conter, Jan Stary and Ester Zapotocka collected the data; Andreas H. Groll, Thomas Lehrnbecher, Simone Cesaro, Andishe Attarbaschi, Daniel Ebrahimi‐Fakhari, Anja Möricke, Martin Schrappe, Martin Zimmermann and Sarah Elitzur analysed the data; Andreas H. Groll, Thomas Lehrnbecher, Simone Cesaro, Andishe Attarbaschi, Daniel Ebrahimi‐Fakhari, Anja Möricke, Martin Schrappe and Sarah Elitzur wrote the manuscript. All authors critically read and discussed the manuscript, and all authors approved the final version of the manuscript.

## FUNDING INFORMATION

The clinical trial AIEOP‐BFM ALL2009 has been supported by grant No. 108588 of the Deutsche Krebshilfe, Bonn, Germany.

## CONFLICT OF INTEREST STATEMENT

Andreas H. Groll has received grants from Gilead, Merck, Sharp & Dohme and Pfizer and has served as consultant to Amplyx, Astellas, Basilea, F2G, Gilead. Merck, Sharp & Dohme, Pfizer, Scynexis and Mundipharma. Simone Cesaro served at the speaker's bureau of Gilead Sciences and Pfizer. Andishe Attarbaschi has received honoraria for lectures, consultancy or advisory board participation from the following companies: Jazz Pharmaceuticals, Amgen, Novartis, MSD, Jazz Pharmaceuticals, Amgen, Novartis, MSD and Gilead. He has received compensation for travel expenses from Jazz Pharmaceuticals. Daniel Ebrahimi‐Fakhari served at the speaker's bureau of Merck/MSD. Anja Möricke has received compensation for travel expenses and honoraria for consultancy from Jazz Pharmaceuticals. Martin Schrappe and/or study group have received research support from SHIRE, JazzPharma, Servier, SigmaTau, Amgen and Novartis. Martin Schrappe has received honoraria from Servier, Novartis and JazzPharma. Thomas Lehrnbecher has received a grant from Gilead Sciences, has served as consultant to Gilead Sciences, Merck/MSD, Pharming, Mundipharma, Pfizer, Recordati Pharma and Roche and served at the speaker's bureau of Gilead Sciences, Merck/MSD, AstraZeneca, Pfizer, Sanofi Pasteur, Recordati Pharma and Mundipharma.

## Data Availability

Under the permission that national data protection requirements are fully met, access to individual data may be made available upon reasonable request to the authors.
